# Left Diaphragmatic Herniation following Orthotopic Liver Transplantation in an Adult

**DOI:** 10.1155/2015/836142

**Published:** 2015-05-04

**Authors:** Adriá Rosat, Ayaya Alonso, Javier Padilla, Pablo Sanz, M. Aránzazu Varona, Javier Méndiz, Enrique Moneva, Manuel Barrera

**Affiliations:** ^1^Division of General Surgery, Department of Surgery, Hospital Universitario Nuestra Señora de Candelaria, Carretera Del Rosario 145, 38010 Santa Cruz de Tenerife, Spain; ^2^Division of Transplantation Surgery Unit, Department of Surgery, Hospital Universitario Nuestra Señora de Candelaria, Carretera Del Rosario 145, 38010 Santa Cruz de Tenerife, Spain; ^3^Transplantation Surgery Unit and General Surgery Service, Hospital Universitario Nuestra Señora de Candelaria, Carretera Del Rosario 145, 38010 Santa Cruz de Tenerife, Spain

## Abstract

Diaphragmatic herniation is an uncommon complication in the postquirurgic follow of the liver transplant. The associated symptoms are unspecific and may not suggest the correct diagnosis. It may explain why in many patients the diagnosis remains unmade or it is made only after a long interval of time. We present the case of a fifty-seven-year-old male who required an orthotopic liver transplant in 2010 due to a trifocal hepatocarcinoma. In postoperatory follow-up the patient showed alimentary regurgitation, vomiting, and dyspepsia. The diagnosis was made by an oesophagogastroduodenal transit with barium and an abdominal CT scan that showed a left diaphragmatic herniation with the gastric fundus into the thorax. With these findings we decided to perform a programmed surgery. After takedown of adhesions and replacement of the stomach into the upper abdomen, the palm-sized diaphragmatic opening was closed with a synthetic material. The patient's condition remained stable throughout the entire operation. The postoperative course was uneventful and he was discharged at the fifth day after surgery with a normal digestive intake. In a 12-month follow-up the patient shows no symptoms.

## 1. Introduction

Diaphragmatic herniation is an uncommon complication in the postquirurgic follow of the liver transplant. Few cases had been described in orthotopic liver transplants in adults [1], being more frequent in child transplants or after partial transplants [2]. The associated symptoms are unspecific and may not suggest the correct diagnosis [3]. It may explain why in many patients the diagnosis remains unmade or it is made only after a long interval of time [4, 5].

## 2. Case Presentation

We present the case of a fifty-seven-year-old male who required an orthotopic liver transplant in 2010 due to a trifocal hepatocarcinoma (Stage A4 of the BCLC, inside Milan criteria) with an alcoholic cirrhosis and hepatitis B (Child's class B (9 points) and a Model for End-Stage Liver Disease score of 18).

**Figure 1:**
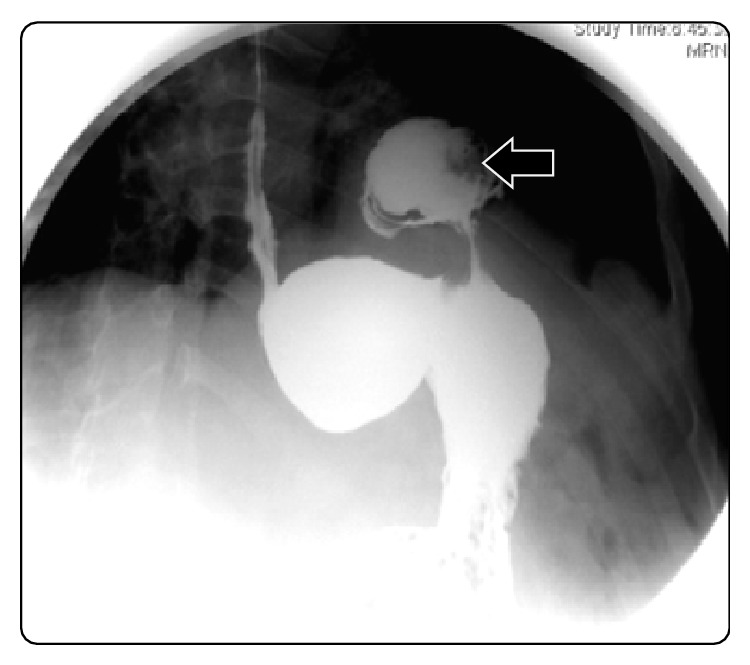
Oesophagogastroduodenal transit (arrow points at gastric content into the thorax).

**Figure 2:**
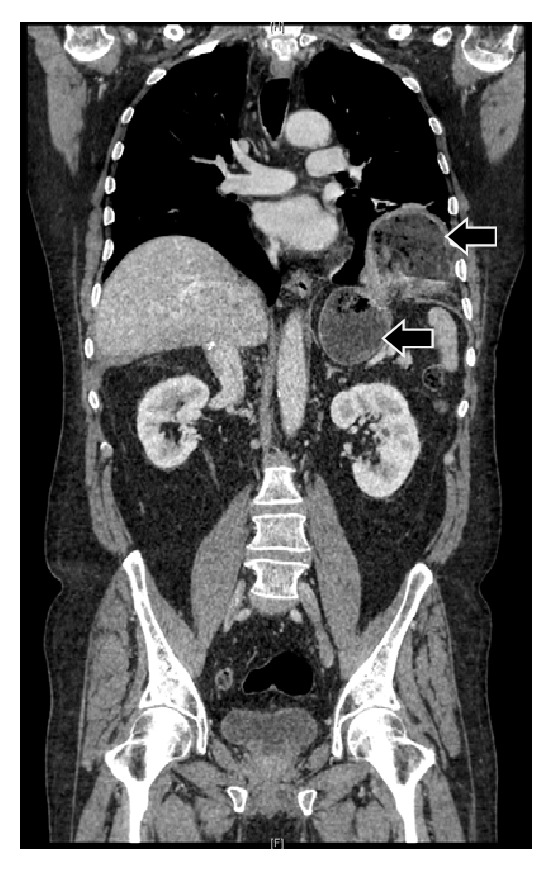
Abdominal CT scan (both arrows point at gastric content, the highest one into the thorax).

Posteriorly he developed a stenosis of the biliary anastomosis that required hepaticojejunostomy in 2011, leading to a small leak which did not require reintervention. Two years later in 2013 he required quirurgic drainage of an abdominal collection. In postoperatory follow-up the patient showed alimentary regurgitation, vomiting, and dyspepsia. The diagnosis was made by an oesophagogastroduodenal transit with barium (Figure 1) and an abdominal CT scan (Figure 2) that showed a left diaphragmatic herniation with the gastric fundus into the thorax.

**Figure 3:**
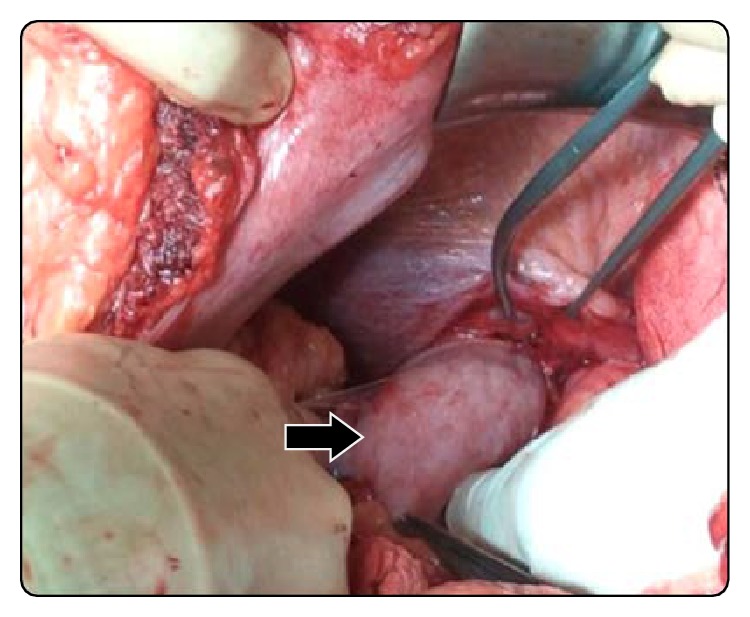
Gastric herniation (photo taken from the left of the patient; arrow points at stomach being pulled back into the abdomen).

**Figure 4:**
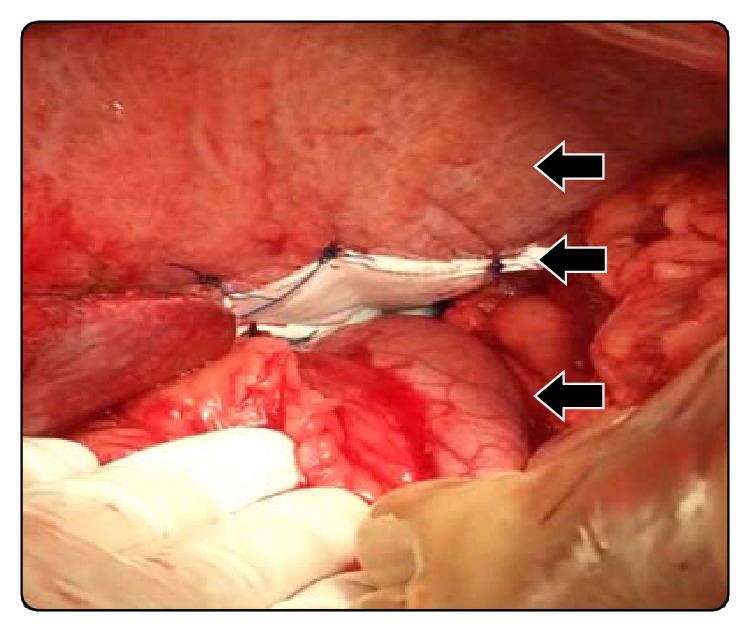
Mesh reparation (photo taken from the left of the patient. Arrows point at, starting from top, the diaphragm, the mesh, and the stomach back into the abdomen).

With these findings we decided to perform a programmed surgery. After takedown of adhesions and replacement of the stomach into the upper abdomen, the palm-sized diaphragmatic opening (Figure 3) was closed with a synthetic material (Figure 4). The patient's condition remained stable throughout the entire operation. The postoperative course was uneventful and he was discharged at the fifth day after surgery with a normal digestive intake. In a 12-month follow-up the patient shows no symptoms.

## 3. Discussion

The responsible mechanism of the muscle rupture after liver transplant may be a result of many factors, such a devitalized diaphragmatic muscle [3], a traumatic dissection, or the excessive use of diathermy during liver transplantation [6].

Ischemia caused by diathermy to the diaphragm during hepatectomy with or without a raised abdominal pressure resulting from an oversized liver graft may also lead to diaphragmatic injury and necrosis, which can progress to herniation. In some cases, a degree of diaphragmatic injury is inevitable with the use of diathermy and suturing when hemostasis is achieved in the presence of diaphragmatic collaterals and portal hypertension [7]. There is a delayed healing of the diaphragm caused by the combination of the constant motion of the diaphragm, local trauma from diathermy, and steroid or other immunosuppressive therapies such as a mammalian target of rapamycin inhibitor impairing healing [8].

One case of spontaneous diaphragmatic rupture has been reported 16 months after liver transplant in a fifty-eight-year-old patient who started to be treated with sirolimus two months after liver transplant for alcohol-related chronic liver disease and hepatocellular carcinoma [9]. Decreased thickness of the diaphragm has been related to a low body weight and malnutrition [2, 6]. Also the patient's pretransplant medical condition may predispose him to the occurrence of posttransplant diaphragmatic herniation, for example, secondary to increased intra-abdominal pressure caused by ascites [6]. All these factors could contribute to a small unseen lesion that will eventually grow causing a herniation.

The symptoms may be minimal and nonspecific, such as colic abdominal pain or digestion problems. In other cases the acute thoracic occupation may cause lung compression and mediastinum displacement leading to respiratory insufficiency and shock. The image study should include chest radiography, abdominal echography, an abdominal CT scan, and an oesophagogastroduodenal transit with barium in order to make the cause of the nonspecific symptoms clear and exclude other complications.

The decision to perform a surgery on a patient with unspecific symptoms is difficult because the origin can be caused by another condition, such as subocclusive adhesions or an altered intestinal motility that may persist after the liver transplant. We suspected an iatrogenic diaphragmatic herniation because in the transplantation surgery in 2010 the patient had hepatomegaly of the left hepatic lobe and a difficult separation from the diaphragm may have caused an unseen injury.

The approach to repair the diaphragm is arguable. The abdominal approach means the mobilization of the graft and bowels, which might be very laborious and harmful. In other cases the thoracic approach, which offers a good and extense exposure of the diaphragm but less control over the abdominal elements, can be preferred. We think that every case has to be taken under consideration separately, evaluating the characteristics of each patient. We decided on an open abdominal approach because of the previous interventions and the possible adherence that may make the hernia reduction through a thoracic approach difficult.

Diaphragmatic hernias present with subtle symptoms but may be a cause of major bowel loss. Surgical repair should be undertaken immediately, and recurrence after the primary repair appears to be uncommon [7]. In our opinion once the diagnosis of the diaphragmatic herniation is made, the patient should be operated on as soon as possible, in order to avoid cardiorespiratory problems or herniated bowels incarceration.
